# Primary Sjogren's Syndrome Presenting as Acute Interstitial Pneumonitis/Hamman-Rich Syndrome

**DOI:** 10.1155/2016/4136765

**Published:** 2016-10-13

**Authors:** Abidullah Khan, Mohammad Humayun, Iqbal Haider, Maimoona Ayub, Zakir Shah, Fahad Ajmal

**Affiliations:** KTH Peshawar, Peshawar, Pakistan

## Abstract

A previously well, 45-year-old Pakistani lady was admitted to the medical unit on-call of Khyber Teaching Hospital (KTH) Peshawar with a 5-day history of fever, productive cough with copious mucoid sputum, dyspnea, and pleuritic chest pain. She also complained of dry eyes, mouth, and vagina. Her chest X-ray showed diffuse alveolar shadowing and arterial gas analysis confirmed type 1 respiratory failure. Over the next few days, she deteriorated rapidly making an urgent transfer to the medical intensive care unit (MICU) necessary, where she was mechanically ventilated. An HRCT followed by bronchoscopic biopsies made a diagnosis of acute interstitial pneumonitis (AIP), formerly known as Hamman-Rich syndrome. She also turned out to be positive for both anti-SS-A/Ro and anti-SS-B/La antibodies along with a positive Schirmer's test and lower lip biopsy. She received intravenous steroids and supportive care. The patient had a complete recovery after approximately three weeks' stay in the hospital with lung function returning back to normal. This is most probably the first ever case of primary Sjogren syndrome (pSjS) presenting as AIP, recovering completely in less than a month time.

## 1. Introduction

Acute interstitial pneumonitis (AIP) is a rare but severe lung disease of unknown etiology that usually affects otherwise healthy individuals. This is also known as acute interstitial pneumonia or Hamman-Rich syndrome [1]. Acute interstitial pneumonitis is notorious for the rapidity with which it progresses, often necessitating hospitalization and mechanical ventilation only days to weeks after initial symptoms of cough, fever, and difficulties breathing develop [2]. To the best of our literature search, this is most probably the first ever case of AIP as the initial presentation of primary Sjogren's syndrome (pSjS), with surprisingly uncomplicated complete recovery in less than a month time, despite a huge mortality rate as high as 70%.

## 2. Case Presentation

**Figure 1:**
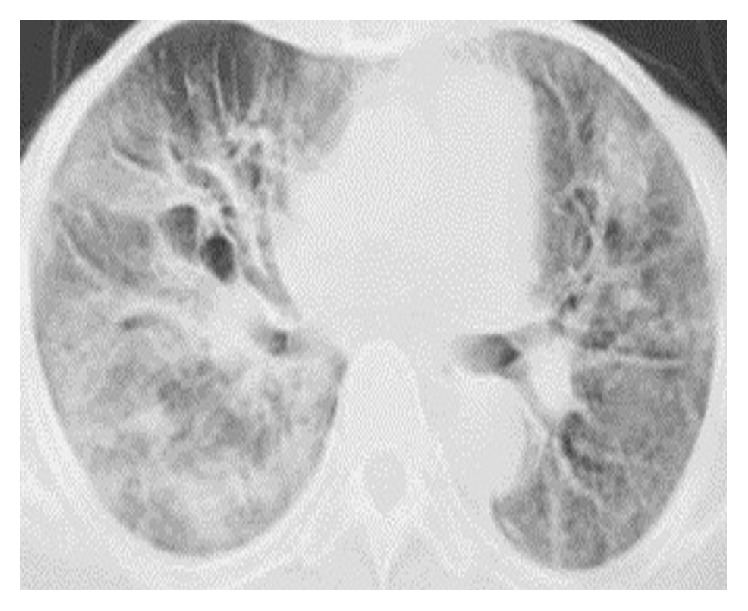
Section of the HRCT chest done on presentation showing bilateral ground glass opacities and a degree of bronchiectasis.

A 45-year-old Pakistani female was admitted to the department of medicine of Khyber Teaching Hospital (KTH) Peshawar with approximately five days' history of high grade fever, pleuritic type chest pain, dyspnea, and productive cough with mucoid sputum. She had been previously fit and well. However, she did have dry eyes, mouth, and vagina, causing dyspareunia over the preceding six months for which she never went to see a doctor. She did not use any regular medications. She had no known exposure to any organic or inorganic dust and usually remained indoors. On clinical examination, she was pyrexial, pale, and tachypneic with oxygen saturation of 92% on room air. Her chest expansion was reduced on both the sides with wide-spread crepitations bibasally. The rest of her examination was completely normal except for dry red eyes, xerostomia, and vaginal dryness with crusting. She was started on oxygen and broad spectrum antibiotics and blood was sent to the laboratory. An immediate bedside chest X-ray showed diffuse alveolar shadowing and type 1 respiratory failure on arterial blood gas (ABG) analysis. A full blood count showed mild anemia with leukocytosis and normal platelets. Her eosinophil count was normal. She had a negative* Aspergillus* precipitins test. She had a normal ECG, echocardiogram, blood sugar, liver, and renal functions. There was no growth of any organism on blood, throat, and urine cultures, respectively. A high resolution CT (HRCT) chest was consistent with ground glass opacities in both the lower zones with some degree of traction bronchiectasis (Figure 1). Considering her complaints of dry eyes and mouth, autoimmune screen was sent which was notable for both positive anti-SSA/Ro and anti-SSB/La antibodies only. She had negative autoantibody test results for other autoimmune diseases like systemic lupus erythematosus (SLE), rheumatoid arthritis (RA), systemic sclerosis (SS), mixed connective tissue disease, and so forth. A flexible bronchoscopy showed normal bronchi with a degree of inflammation distally. Multiple endoscopic bronchial biopsies were taken. The biopsies were consistent with diffuse alveolar damage (DAD). Bronchoalveolar lavage (BAL) based polymerase chain reaction (PCR) for respiratory viruses, namely, influenza, parainfluenza, and respiratory syncytial virus (RSV),* Mycobacterium tuberculosis*, and bacterial cultures were all unremarkable. A lower lip biopsy showed sialadenitis with focal lymphocytic infiltrate. Schirmer's test was positive with only 3 cm of wetness after five minutes' insertion in the conjunctival sac.

**Figure 2:**
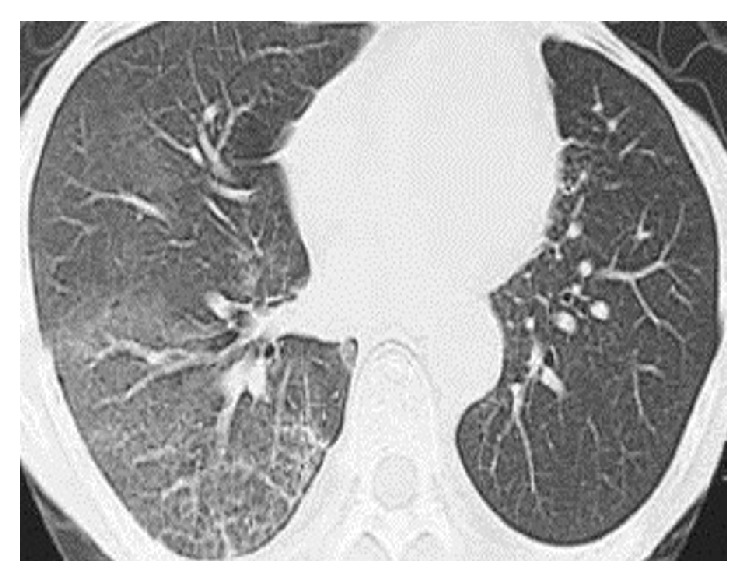
A section of HRCT done prior to discharge showing dramatic improvement in the lung filed shadowing.

On the 4th day of admission, the patient deteriorated rapidly and was shifted to the medical ICU where she was intubated and mechanically ventilated. She received three days' course of intravenous methyl prednisolone to which she had a dramatic response. She was extubated on the fifth day of her admission to the medical ICU and was continued on intravenous dexamethasone 8 mg three times and piperacillin-tazobactam (Tazocin) 4.5 gm four times daily, respectively. She was downstepped to the general medical ward after roughly ten days' stay in the ICU. A repeat HRCT chest was remarkably improved with only minimal degree of bronchiectasis and mild alveolar opacities remaining (Figure 2). She had normal ABGs and excellent results on lung functions testing prior to her discharge from the hospital. She was discharged on the tapering regimen of two months' course of oral prednisolone along with artificial saliva and tear drops.

**Figure 3:**
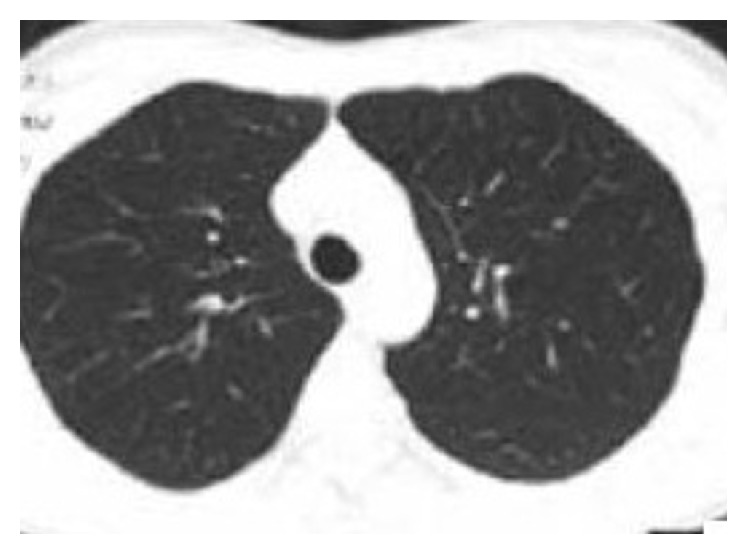
A normal HRCT chest obtained 2 months after discharge.

She was reviewed in the medical OPD a month after her discharge and was in good health with a normal chest X-ray, ABGs, and pulmonary function tests (PFTs). On her 2nd follow-up visit two months later, she continued to be in good health. A repeat HRCT of the chest was normal (Figure 3). We are planning to review her again in 6 months' time with pulmonary function test (PFTs), another chest X-ray, and/or HRCT of the chest.

## 3. Discussion

Sjogren syndrome is a chronic autoimmune disorder primarily affecting the exocrine glands of the body. It is called primary or secondary Sjogren syndrome depending upon the absence or presence of other autoimmune disorders like rheumatoid arthritis and so forth, respectively [3]. Primary Sjogren's syndrome can affect extraglandular organ systems including the lungs. Wide-spread interstitial lung disease is the most dreadful form of lung involvement which can manifest as acute respiratory failure [4]. It is worth mentioning that although screening of asymptomatic patients of Sjogren syndrome with computed tomography, pulmonary function tests, and bronchoalveolar lavage can detect pulmonary involvement in up to 75% of patients, clinically remarkable lung involvement affects roughly 9–24% of patients and may be the first manifestation of this disease [5–7].

Sjogren syndrome can be diagnosed using the new criteria proposed by the American College of Rheumatology (ACR) in 2012 which requires two of the following to classify someone as having Sjogren syndrome: (1) positive serum anti-SSA/Ro and/or anti-SSB/La (or positive rheumatoid factor and anti-nuclear antibodies at a dilution >1/320); (2) minor salivary gland biopsy exhibiting focal lymphocytic sialadenitis; and (3) keratoconjunctivitis sicca with ocular staining score >3 (assuming that the individual is not currently using daily eye drops for glaucoma and has not had corneal surgery or cosmetic eyelid surgery in the past 5 years) [8]. It is notable that our patient fulfilled these diagnostic criteria recommended by ACR.

Sjogren syndrome being a multisystem autoimmune disease can affect the entire respiratory tract, with manifestations ranging from obstructive small airway disease, xerotrachea and bronchial sicca, lung cysts, pulmonary amyloidosis, pulmonary hypertension, lymphoinfiltrative or lymphoproliferative lung disease, and pleural involvement to various patterns of interstitial lung disease (ILD) like nonspecific interstitial pneumonia (NSIP) or usual interstitial pneumonia (UIP). Our patient had clinical, radiological, and histological evidence of acute interstitial pneumonitis (AIP) as the initial presentation of pSjS [9].

Acute interstitial pneumonia (Hamman-Rich syndrome) is an idiopathic, rapidly progressive, and often a lethal form of interstitial lung disease. AIP resembles ARDS and presents as rapidly progressive cough, fever, and phlegm production, leading to respiratory failure in days' to weeks' time needing mechanical ventilation [10, 11]. Histologically AIP is characterized by diffuse alveolar damage (DAD) which along with rapid clinical progression of the disease is a hallmark feature [12, 13]. Our patient had both of these clinicopathological features.

AIP has a bad prognosis with mortality approaching 50–70%; however, those who survive the initial period of the disease may have a good long term survival and rarely a complete recovery [14]. Our patient remained on ventilator for roughly a week and received intravenous methyl prednisolone along with supportive treatment and despite a very bad CT chest on presentation had a complete recovery.

The diagnosis of AIP demands a thorough clinical assessment; however, biopsy is the criterion standard despite its difficulties. The biopsy usually shows acute or organizing diffuse alveolar damage (DAD). Both transbronchial and open lung biopsies can be obtained. However, before proceeding to open lung biopsy, multiple endoscopic bronchial biopsies during bronchoscopy seem to be the first logical choice. The radiological findings on high resolution CT (HRCT) chest include bilateral ground glass opacities and consolidation of the lungs predominantly in the lower zones. In the later stages HRCT shows traction bronchiectasis and fibrotic changes [5, 12, 15]. The initial HRCT of our patient showed bibasal consolidation and ground glass opacification along with patchy traction bronchiectasis; however, the repeat HRCT on discharge was remarkably improved with only few areas of bronchiectasis remaining.

Treating Sjogren syndrome and its pulmonary manifestations can be challenging. The management of tracheobronchitis and bronchiectasis needs treatment for dryness and/or inflammation of the airways. Moreover, corticosteroid therapy is the mainstay of pulmonary interstitial disease's treatment in Sjogren's syndrome, but the use of other immunosuppressive drugs like azathioprine, cyclophosphamide, and so forth needs to be determined, especially if the chronic form of lung involvement develops [9]. Our patient showed dramatic response to three days' course of intravenous methyl prednisolone followed by a week course of dexamethasone. She was discharged home on tapering regimen of two months' course of oral prednisolone given initially at 1 mg/kg body weight. She was reviewed 8 weeks after she was discharged from the hospital and was stable with regard to her lung disease with a normal X-ray chest and PFTs; however, she still had dry eyes and mouth for which she is using artificial tears and saliva, respectively.

## 4. Conclusion

Acute interstitial pneumonitis (AIP) is a rare, but potentially lethal, complication of Sjogren syndrome and can be the initial presentation of the disease per se. Management can be very difficult, often necessitating mechanical ventilation and intravenous steroids; however, those surviving the acute phase can recover completely with a good long term prognosis.
